# Highly Pathogenic Avian Influenza A(H5N1) Virus: How Far Are We from a New Pandemic?

**DOI:** 10.3390/vetsci12060566

**Published:** 2025-06-09

**Authors:** Giovanni Di Guardo

**Affiliations:** General Pathology and Veterinary Pathophysiology, Veterinary Medical Faculty, University of Teramo, Località Piano d’Accio, 64100 Teramo, Italy; gdiguardo@unite.it

**Keywords:** highly pathogenic avian influenza A(H5N1) virus, cow H5N1, cattle H5N1, pandemics, pandemic risk, emerging infectious diseases, zoonoses, susceptible hosts’ range, birds, mammals

## Abstract

The focus of this commentary is represented by the pandemic risk associated with the highly pathogenic avian influenza (HPAI) A(H5N1) virus, clade 2.3.4.4b. More in detail, the herein dealt pandemic alarm appears to be primarily justified by the huge and progressively growing number of virus-susceptible domestic and wild birds and mammals, including threatened marine mammal species like South American sea lions and elephant seals as well as harbour porpoises, bottlenose dolphins and polar bears. Of major concern is the susceptibility of dairy cattle to HPAI A(H5N1) virus, particularly the documented and unprecedented colonization of host’s mammary gland tissue, resulting in viral shedding through the milk alongside a large series of cases of infection in dairy farm workers in several USA locations. Despite well-documented zoonotic capability, no evidences of a sustained and efficient HPAI A(H5N1) viral transmission between people have been hitherto reported. If this were to happen sooner or later, a new pandemic might consequently arise. Therefore, keeping all this in mind and based upon the lessons taught by the COVID-19 pandemic, a “One Health, One Earth, One Ocean”-centered approach would be absolutely needed in order to deal in the most appropriate way with the HPAI A(H5N1) virus-associated zoonotic and pandemic risk.

## Simple Summary

The present commentary deals with the pandemic risk brought about by the highly pathogenic avian influenza (HPAI) A(H5N1) virus. Such a pandemic alarm is justified, among others, by the progressively and rapidly expanding range of virus-susceptible hosts, including humans alongside a significant number of domestic and wild avian and mammalian species, several of which appear to be geographically and phylogenetically distant from each other. Despite its well-established zoonotic potential, no clear-cut evidence of a sustained and efficient HPAI A(H5N1) virus interhuman transmission has thus far been reported. Should this happen in a more or less near future, it could pave the way for a new pandemic. Based upon the above, a “One Health, One Earth, One Ocean”-focused, holistic approach would be the necessary prerequisite to deal in an appropriate way with the HPAI A(H5N1) virus-associated zoonotic and pandemic risk.

Since its emergence as a novel reassortant virus from subtype A(H5N8), the highly pathogenic avian influenza (HPAI) A(H5N1) virus of clade 2.3.4.4b has been responsible for a large number of outbreaks worldwide in both wild and domestic avian and mammalian species, a significant proportion of which also appear to be geographically and phylogenetically distant from each other. Compared to its parental A(H5N8) clade 2.3.4.4b, the novel reassortant HPAI A(H5N1) virus has demonstrated an increased ability to cross species barriers, thus infecting multiple mammalian species, including humans. This is the main reason why such a viral pathogen is increasingly perceived as a global threat [1,2]. Following its first identification in 1959 in Scottish poultry farms [3], the HPAI A(H5N1) virus was detected in 1996 in domestic waterfowl in Southern China, with the agent being named A/goose/Guangdong/1/1996. In 1997, the first 18 human cases, 6 of which were lethal, occurred in Hong Kong in close epidemiological association with HPAI A(H5N1) poultry outbreaks. Additionally, since 2005, wild birds spread the virus to poultry in Africa, the Middle East, Europe and, thereafter, also to domestic and wild avian and mammalian species in North and South America, with the viral haemagglutinin (HA) gene giving rise to multiple genetic groups (clades) of H5 viruses displaying various neuraminidase (NA) genes, including the herein dealt HPAI A(H5N1) virus clade 2.3.4.4b [4,5].

Within such a framework, the recent migratory bird-driven spread of HPAI A(H5N1) virus clade 2.3.4.4b to a huge and progressively expanding number of domestic and wild avian and mammalian host species from Eurasia, North and South America, up to the Arctic and Antarctica regions—including sea lions (*Otaria flavescens*) and elephant seals (*Mirunga leonina*) along the Pacific and Atlantic coasts of South America—is of concern [6,7] according to the scientific community, the World Health Organization, the World Organization for Animal Health and the Public Health Authorities from many European and extra-European countries. As a matter of fact, the aforementioned pinnipeds are included in the International Union for the Conservation of Nature (IUCN) Red List of Threatened Species, along with other virus-susceptible marine mammal species like polar bears (*Ursus maritimus*) [8], harbour porpoises (*Phocoena phocoena*) [9] and bottlenose dolphins (*Tursiops truncatus*) [10].

As far as cases in people are specifically concerned, the World Health Organization (WHO) has recently published data on the cumulative number of reported and confirmed human cases of HPAI A(H5N1) virus infection in 24 countries, including 964 cases with 466 deaths, resulting in a mortality rate of 48% [11]. In this respect, although no clear-cut evidence of sustained and efficient viral interhuman transmission has been hitherto reported, there is increasing concern that infections in poultry and cattle with transmission to humans are growing due to inadequate control measures. Among the four influenza virus types, A, B, C and D, cattle are most susceptible to influenza D infection, additionally acting as a *reservoir* for this seven-segmented influenza virus. And, although cattle susceptibility to type A influenza viruses had already been shown [12], the recent outbreaks of the HPAI A(H5N1) virus among dairy cows in the USA, where cattle-to-cattle transmission has also been reported [1], were largely unpredictable. To date, this viral pathogen has spread into 14 states, affecting 299 dairy herds and causing clinical symptoms such as reduced appetite, fever and a sudden drop in milk production. Infected cows can also transmit the virus through raw, unpasteurized milk [11]. Surprisingly enough, however, no additional cases have thus far been ascertained in cattle from countries other than the USA, while a case of A(H5N1) viral infection has recently been described in a sheep in the United Kingdom [13]. Similar to the avian A(H5N1) clade 2.3.4.4b, the cattle-infecting virus may also transmit to humans—dairy farm workers in particular, who are typically infected through milk splash events—as well as to contact animals like cats, raccoons, rodents, opossums and poultry [1]. As an example of infection in animals, following the consumption of unpasteurized milk from infected cows, in which viral replication appears to be mainly restricted to mammary gland tissue, cats may develop a severe respiratory and neurological disease [14]. This seems to be quite different from what is generally observed in virus-infected farm workers, who often exhibit a bilateral conjunctivitis sometimes accompanied by a febrile syndrome with mild respiratory signs [15]. Within such a challenging and fast evolving *scenario*, a major concern is raised by the fact that this epidemic in cattle represents the largest A(H5N1) viral outbreak in a domestic mammal close to humans, thus enhancing the risk that a mammalian-adapted agent gives rise, following the acquirement of *ad hoc* genetic mutations, to a sustained and efficient interhuman transmission chain ultimately resulting in a new pandemic [1]. Furthermore, since the bovine mammary gland may be targeted by both avian-type and human-type A(H5N1) viruses, this might contribute to “accreditate” cattle as an additional “mixing vessel”, allowing their genetic reassortment and recombination in a similar fashion to the swine species as far as concerns the evolution and transmission pathways of influenza viruses between birds and people [16]. Additional alarming issues related to the potential development of a “pandemic behaviour” on behalf of the HPAI A(H5N1) virus are undoubtedly represented by the frequently occurring mutations involving its genetic make-up, with special emphasis on HA, NA and polymerase (P) genes, leading in turn to viral genomic shifts and antigenic drifts. In this respect, a number of mutational events increasing viral replication in mammals as well as in mammalian cell lines have already been identified, including the well-established PB2 E627K mutation linked to enhanced virulence [17] alongside the newly discovered HA Q226L substitution switching bovine A(H5N1) viral tropism toward human-type rather than avian-type receptors [18].

The prominent viral neurotropism and neuropathogenicity, clearly documented by the severe encephalitic and meningo-encephalitic lesions found in HPAI A(H5N1) virus clade 2.3.4.4b-affected humans, cats and marine mammals [8,9,10,11,14,19,20], coupled with a high infection lethality rate, should be regarded as further relevant concern issues, especially in view of the potential virus shift toward a pandemic behaviour.

Noteworthy, despite the hitherto available “absence of evidence” in favour of a sustained and efficient HPAI A(H5N1) virus clade 2.3.4.4b interhuman transmission, the recently demonstrated susceptibility of mice and ferrets to a viral strain recovered from the conjunctiva of a patient in Texas should also be regarded as a finding of particularly significant importance in relation to the putative development of a viral pandemic behaviour. Such experimentally challenged animals displayed, in fact, a lethal systemic infection characterized by severe bilateral pneumonia and encephalitis [21]. Notwithstanding the above, however, a recent study has also reported that a human HPAI A(H5N1) virus isolate of bovine origin binds poorly to human-type sialic acid receptors, an apparently reassuring finding [22].

Finally, since the global spread of HPAI A(H5N1) virus poses a serious pandemic threat, the swift development of effective human and animal vaccines should be viewed as a top priority, although we would be profoundly mistaken whenever thinking this should be the only action to be undertaken for efficiently tackling the potential start of a new pandemic. To this aim, the success of messenger RNA (mRNA) technology-based vaccines in efficiently counteracting the COVID-19 pandemic demonstrates its potential for addressing other infectious threats in people as well as in animals, such as the herein dealt HPAI A(H5N1) viral clade 2.3.4.4b. This has been clearly shown, for example, in ferrets previously immunized with *ad hoc* mRNA vaccines against viral HA and NA antigens, which were subsequently challenged with a lethal dose of HPAI A(H5N1) virus [23].

As a concluding remark, it is my strong belief that we should firmly keep in mind, first of all, the memorable and remarkable lessons taught by the COVID-19 pandemic and, more in general, by past infectious disease outbreaks caused by zoonotic pathogens, as it happened over one century ago with the catastrophic “Spanish flu” pandemic. Indeed, while it seems undeniable that the history of mankind, along with that of all living organisms, has been continuously shaped by infectious agents, within complex host–pathogen coevolutionary dynamics, it should also be emphasized that the origin of “emerging infectious diseases”, either proven or suspect, lies for two thirds of them in one or more animal *reservoirs* [24]. In consideration of the above, the “One Health, One Earth, One Ocean” paradigm, reminding us that human, animal and environmental health are mutually and inextricably linked to each other, should be regarded as the leading principle and the necessary prerequisite upon which we should develop all the actions and strategies for efficiently tackling and counteracting HPAI A(H5N1) virus spread among birds and mammals as well as from birds and mammals to humans (and *vice versa*) and, most importantly, between human beings, with the aim of avoiding a future pandemic. Among such actions and strategies, the following ones should be viewed as top priorities: (a) building a tight collaboration between human and animal health sectors; (b) improving eco-epidemiological surveillance for early detection of human infectious disease threats; (c) strengthening laboratory diagnostic capacities for novel microbial pathogens; (d) developing epidemic/pandemic preparedness for and response to emerging infectious agents; (e) tracking and communicating the appearance and subsequent spread among people and animals of new genetic mutants and/or reassortants of previously known pathogens; (f) developing and employing *ad hoc* risk analysis protocols related to infectious disease threats; (g) setting up *ad hoc* vaccines and immunization protocols [25].

Within such a challenging, complex and largely unpredictable *scenario*, it is hard to believe that the “COVID-19 Scientific Committee”, popularly known by the acronym “CTS” (standing for “Comitato Tecnico-Scientifico”) and which was officially set up in February 2020 by the Italian government for counteracting the COVID-19 pandemic, was inexplicably dismantled two years later, with no veterinarians having ever been co-opted in it. How useful, by contrast, would have been a similar “organism” (duly revisited in its composition, thus including *ad hoc* veterinary medical expertise) for efficiently tackling, preventing, predicting, quantifying, handling and communicating the risk of future epidemic and pandemic human and animal disease outbreaks [26]!

*Historia magistra vitae* and, no less important, *Errare humanum est perseverare autem diabolicum*!

## Data Availability

Not applicable.
